# Impact of the COVID-19 pandemic on surgical education and training programs in Latin America: a systematic review

**DOI:** 10.3389/fmed.2024.1499436

**Published:** 2024-11-20

**Authors:** Felipe Loza Hernandez, Pamela Ochoa Lantigua, Vanesa Puga Rosero, Sebastian Jara Jimenez, Mateo Carrera Cajamarca, Jose E. Leon-Rojas

**Affiliations:** ^1^Faculty of Health and Life Sciences, Medical School, Universidad Internacional del Ecuador, Quito, Ecuador; ^2^Escuela de Medicina, Universidad de las Américas (UDLA), Quito, Ecuador

**Keywords:** COVID-19, medical education, impact, surgical education, resident

## Abstract

**Background:**

The COVID-19 pandemic posed challenges to surgical training, demanding a balance between theoretical and practical knowledge, especially in Latin American countries. Therefore, our aim was to characterize the challenges faced by surgical education in these countries.

**Methods:**

A systematic search was conducted on PubMed, Scopus, and the Virtual Health Library on March 23, 2022, yielding 2,838 articles. Articles were filtered by two independent reviewers focusing on the effect of the pandemic in surgical education.

**Results:**

A total of 31 articles were selected; 54.83% of surgical programs reported a reduction in surgical involvement by trainees. First-year residents were the most affected, with some surgical residency programs forced to shift their residents to take care of COVID-19 patients; additionally, in 67.74% of cases, online courses and virtual simulation was implemented. Most of the residents had a positive opinion regarding virtual lectures but considered that their surgical skills were affected.

**Conclusion:**

The development of surgical skills was hindered by changes in surgery prioritization, techniques, and a decrease in caseload. The pandemic also caused a reliance on virtual formats for education and patient care. This shift created irregularities in training but increased opportunities for alternate activities.

## Introduction

SARS-CoV-2, the virus responsible for COVID-19, was declared a pandemic in March 2020 and endured for almost two and a half years causing significant effects worldwide. Medicine and patient care were significantly impacted, particularly in terms of the training of future healthcare professionals (1). The medical educational system had to adjust to isolation and innovate new techniques to ensure continued education, including modifying teaching approaches, enhancing the quality, and increasing the quantity of lectures delivered in virtuality (1).

Successful surgical resident training necessitates a combination of theoretical and practical knowledge. Amidst the COVID-19 pandemic, surgical education suffered a significant setback, prompting numerous countries to make necessary adjustments to provide proper training for their medical residents (2). Latin American countries face resource constraints in their healthcare systems, which significantly impacted the ability of hospitals and universities to swiftly adapt to the pandemic and maintain quality healthcare for patients while ensuring adequate training for medical students (2).

Multiple studies have been conducted on the various approaches taken by countries, hospitals, and universities to impart both theoretical knowledge and surgical techniques to their residents. However, there is a scarcity of comprehensive systematic reviews that consolidate this information to provide a global perspective on these developments, particularly in Latin American countries. Therefore, the purpose of our systematic review is to examine the effects of the COVID-19 pandemic on education in several surgical disciplines in Latin American countries to identify the primary challenges faced and the corresponding solutions implemented by the affected institutions.

## Methods

This systematic review followed the Preferred Reporting Items for Systematic Reviews and Meta-Analysis (PRISMA) 2020 guidelines (3).

### Eligibility criteria

We included all articles referring to medical education in surgical residencies in Latin American countries during COVID 19, from the beginning of the pandemic until March 2022. We decided to include the following surgical residency programs: general, cardiothoracic, maxillofacial, orthopedic, pediatric, plastic, neurosurgery, vascular, dermatology, ophthalmology, gynecology, otolaryngology, and urology. Case reports, case control studies, cohorts, randomized trials, commentaries, letters to the editor, perspectives, and “how I do it” articles were included in this review; only papers published in English or Spanish were selected. We excluded articles that did not analyze the effect of the COVID-19 pandemic or that included non-surgical residency programs; we also excluded systematic, scoping or literature reviews, and conference abstracts.

### Information sources and search strategy

Medline (PubMed), Scopus, and the Virtual Health library were searched from 2019 until March, 2022 utilizing medical subheadings (MeSH or DeCs), relevant field operators, and text words. Representative keywords (surgical residency, surgical training, virtual learning, COVID-19, and Latin American countries) were combined with Boolean operators; the complete search strategy for each database can be found in the Appendix.

### Data management

All of the articles retrieved from the three databases were uploaded to the online software Ryyan in order to reduce errors in data entry, article selection, and deduplication.

### Selection process

After the deduplication process, two independent and blinded reviewers screened, in Ryyan, all titles and abstracts applying the aforementioned eligibility criteria; any discrepancies between these authors were solved by a third reviewer. Articles were then screened a second time for eligibility by assessing the full-text by two independent and blinded reviewers in a similar fashion as in the first filtering process.

### Data items

The following data was extracted in a Microsoft Excel spreadsheet: author, year of publication, DOI, changes within the surgical service, changes of specialty, changes in internships, telemedicine use, changes in lectures and conferences, complimentary educational activities, simulators, and personal satisfaction.

### Data synthesis

Data placed in the Excel spreadsheet was analyzed using descriptive statistics in order to determine the relative and absolute frequencies of each of the aforementioned categories related to changes in surgical education (service shift changes, changes in surgeries, changes in lectures, use of telemedicine, use of complementary activities or simulation, and resident satisfaction).

### Bias assessment

Bias assessment was conducted by two-independent and blinded reviewers using the National Heart, Lung, and Blood Institute (NHLBI) Study Quality Assessment Tools (4). Studies were graded as having either minimally low, moderately low, or high risk of bias. When answering yes to 80% or more of the questions, then the study was graded as having a minimally low risk of bias; if answering yes to 50-79% of the questions, then the study was graded as having a moderately low risk of bias; finally, if answering yes to <50% of the questions, then the study was graded as having a high risk of bias. Any discrepancies on grading were solved by a third reviewer and mutual consensus.

## Results

**Figure 1:**
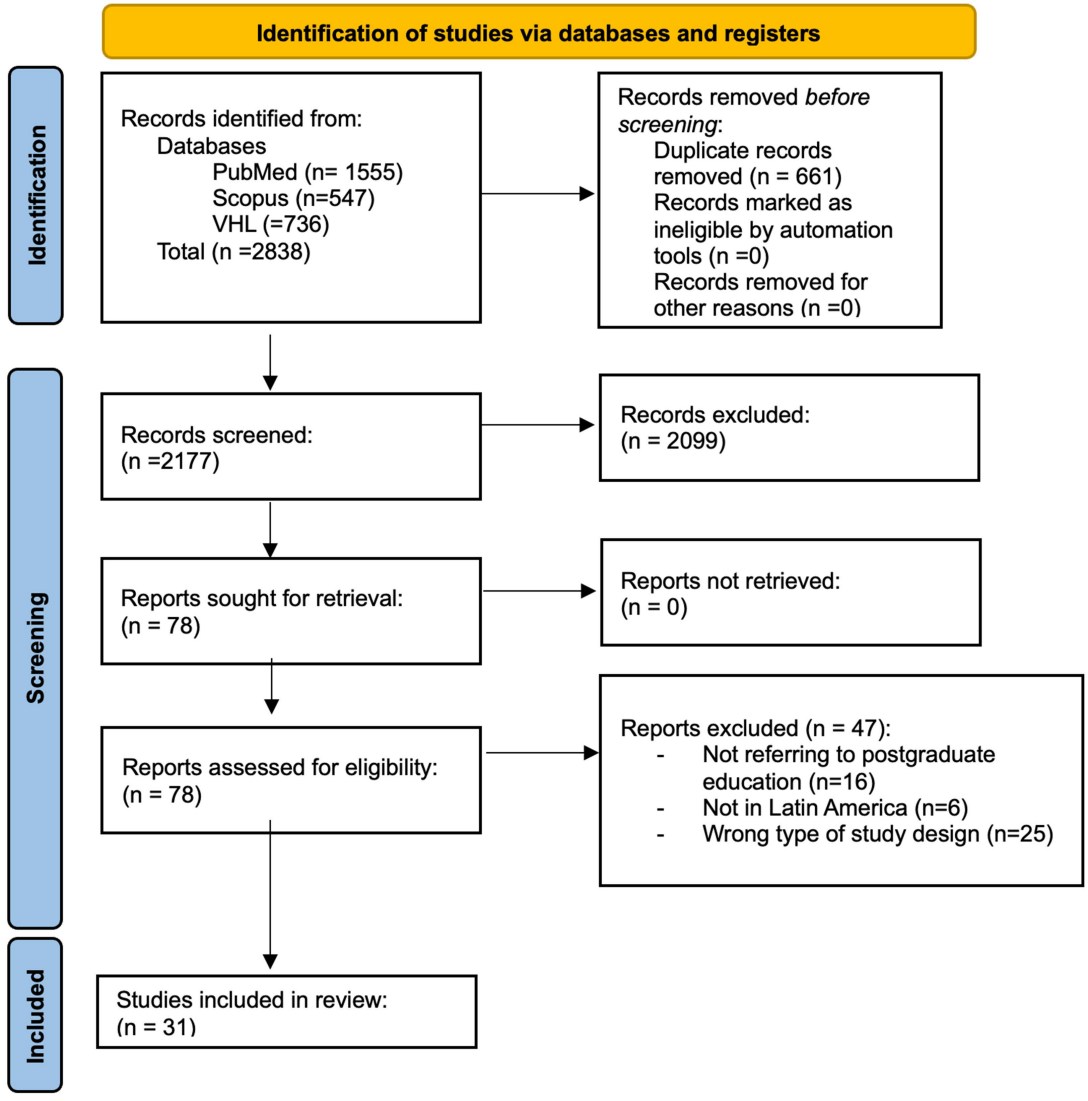
Article selection process (PRISMA flowchart, 2020).

We identified a total of 2,177 articles, after duplicate removal; of those, 78 articles passed the title, abstract, and keyword screening and were subjected to full-text assessment against our eligibility criteria. Finally, 31 articles were included in our review (2, 5–34); the full selection process can be found in Figure 1.

The results of bias assessment can be found in Table 1; in general, 68 and 32% of articles were graded as high and moderate risk of bias, respectively.

**Table 1:** Bias assessment of included studies.

No	Study	Year	Study design	Risk of bias
1	Alvarez M	2020	Letter to editor	High
2	Alvarez M	2021	Cross sectional	High
3	Arenas-Soto	2021	Letter to editor	High
4	Brito L	2020	Letter to editor	High
5	Cabrera, L	2021	Letter to editor	High
6	Chatziralli, I	2021	Cross sectional	High
7	Cho M	2021	Cross sectional	High
8	De la Cerda-Vargas M	2021	Cross sectional	High
9	De la Cerda-Vargas M	2022	Cross sectional	High
10	Diaz Castrillón C	2021	Cross sectional	High
11	Diaz Castrillón C	2020	Cross sectional	High
12	Falcioni A	2022	Cross sectional	High
13	Figueroa F	2020	Cross sectional	High
14	Gondim M	2021	Cross sectional	High
15	Gonzales-Urquijo M	2021	Cross sectional	High
16	Gorgen A	2021	Cross sectional	High
17	Huamanchumo-Suyon M	2020	Cross sectional	High
18	Leite Ana Kober	2021	Cross sectional	Intermediate
19	Lerendegui Luciana	2021	Cross sectional	Intermediate
20	Lima D	2020	Cross sectional	High
21	Munjal Tina	2020	Cross sectional	High
22	Oropeza-Aguilar Mariano	2022	Cross sectional	Intermediate
23	Paesano Nahuel	2020	Cross sectional	Intermediate
24	Pagotto Vitor	2020	Cross sectional	Intermediate
25	Palacios Huatuco Rene	2021	Cross sectional	Intermediate
26	Pawlak Katarzyna	2020	Cross sectional	High
27	Prezotti	2021	Cross sectional	Intermediate
28	Rivera-Chavarria	2021	Cross sectional	Intermediate
29	Rodriguez Santos	2021	Cross sectional	Intermediate
30	Santos	2021	Cross sectional	Intermediate
31	Trujillo	2021	Cross sectional	High

### Reassignments

Several surgical residency programs were forced to reassign their residents to non-surgical departments during the COVID-19 pandemic, as documented in seven studies (2, 8, 12, 20, 22, 26, 35). A survey conducted among neurosurgery residents in Latin America and Spain revealed that 76% of the participants were required to handle COVID-19 patients, with just 57% of them receiving supervision for this obligation (12). Similarly, head and neck surgery trainees in Brazil reported that 11% of them were fully reassigned from their surgical duties to care for COVID-19 patients; out of the 36, one trainee dedicated 75% of their time to COVID-19 patients, two trainees dedicated 50% of their time, and four trainees dedicated only 25% of theirs (22). In a separate poll including urology residents from Ibero-American countries, 15% of respondents reported that their medical facility had to temporarily shut down in order to handle COVID-19 patients. Additionally, some participants had to alternate with their colleagues to carry out administrative tasks (2). During the pandemic, 66.7% of surgical residency centers in Mexico City had to shift their focus exclusively to COVID-19 care to meet the health demands (26). Three further articles documented the reassignment of their surgical residents to non-residency related tasks, due to their aid being required for COVID-19 triage or in the intensive care unit (8, 20, 35).

### Impact in surgical training

We found that surgical residencies over Latin America were affected by significant changes due to the COVID-19 pandemic, and their impact on the surgical resident’s perception or abilities was negative, in general; around 54.83% (*n* = 17) (2, 5–8, 13, 20, 23, 26–31, 33, 36) of programs reported reduction in surgical skills because of a reduction in surgeries, while 0.3% (*n* = 1) reported an increase in surgical training (31). In general, the first-year residents were the most affected (7, 8, 27, 35). We also noted a decrease in the overall number of surgeries in surveys that assessed pre-pandemic counts in comparison to post pandemic cases (20, 23, 27, 28, 31). However, counterintuitively, Rivera Chavarria and colleagues reported an increase in their resident’s surgical skills during the COVID-19 pandemic in Costa Rica. It’s important to note that this was not due to an increase in the number of surgeries, but rather due to an increase in the resident’s participation due to the limited availability of staff; they reported an overall decrease in performed surgeries (from 291 surgeries to 241) but an increase in surgical skills thanks to higher surgical workload (PGY2 residents’ participation in procedures increased from 19 to 27%). They were also allowed to work on more emergency cases (increasing from 40 to 53.3%) and oncological cases (increasing from 14.8 to 21.9%) (31).

**Figure 2:**
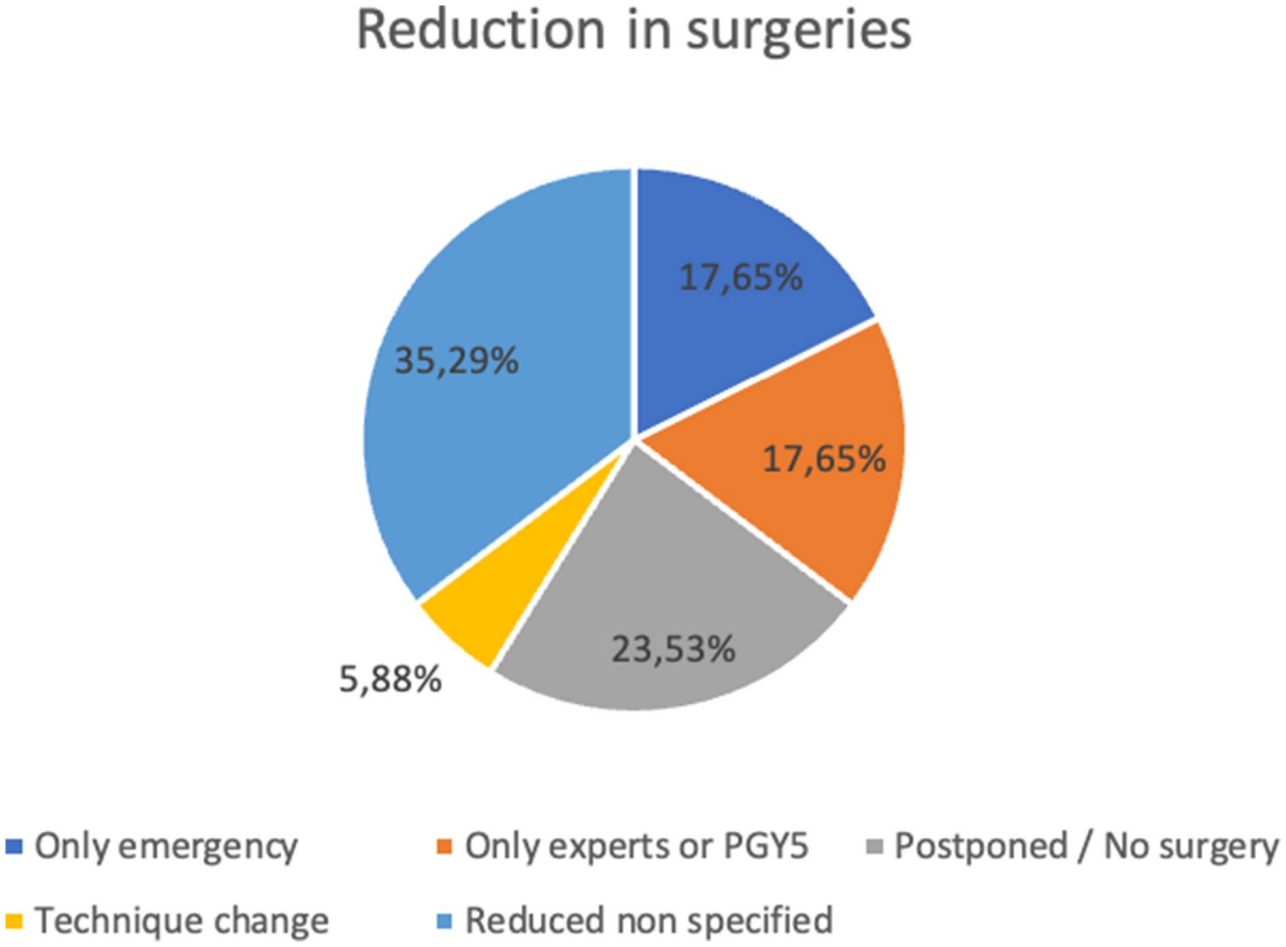
The impact of COVID-19 on surgery frequency in residency.

We identified five categories in which changes were reported: only emergency surgeries were performed, only experts or PGY5 were allowed to operate, surgeries were postponed or canceled, surgical technique changed to avoid risk of transmission, and overall reduction in surgeries with no specific cause. The distribution of these categories can be found in Figure 2.

### Telemedicine

Telemedicine played an important role during the COVID-19 pandemic as face-to-face patient care was limited or impossible to practice. Four of our included surgical programs reported the use of telemedicine for remote patient care and follow-up (2, 7, 14, 26). Only two of the four programs reported using telemedicine for the patient’s postoperative follow-up (7, 14). In one of them, 335 participants, including surgical residents, general surgeons, and subspecialists in Colombia, responded to a survey, and 54.9% of them reported the use of telemedicine for postoperative follow-up, with 78.8% of subspecialists and 48.7% of general surgeons stating that telemedicine is not sufficient as a follow-up method (7).

In the other three programs, the number of residents that used telemedicine to provide remote medical consultations is not specified, as it is only reported to be used due to a reduction of daily hospital workload (2, 7, 26). In a survey in Colombia of 122 dermatology residents, an increase from 5 to 84% of telemedicine consultations was reported; whereas, in a survey of 453 residents in Mexico City, only 7% reported to have kept a normal hospital work routine, and the rest described the use of telemedicine for most of patient care (the exact number of telemedicine consultations is not specified) (7). Finally, in an Ibero-American Urology residents survey, 90% presented changes in their clinical activities, stating the use of telemedicine and telephone calls both for consultations and postoperative follow-up, without specifying an exact number (14).

### Changes in lectures

**Figure 3:**
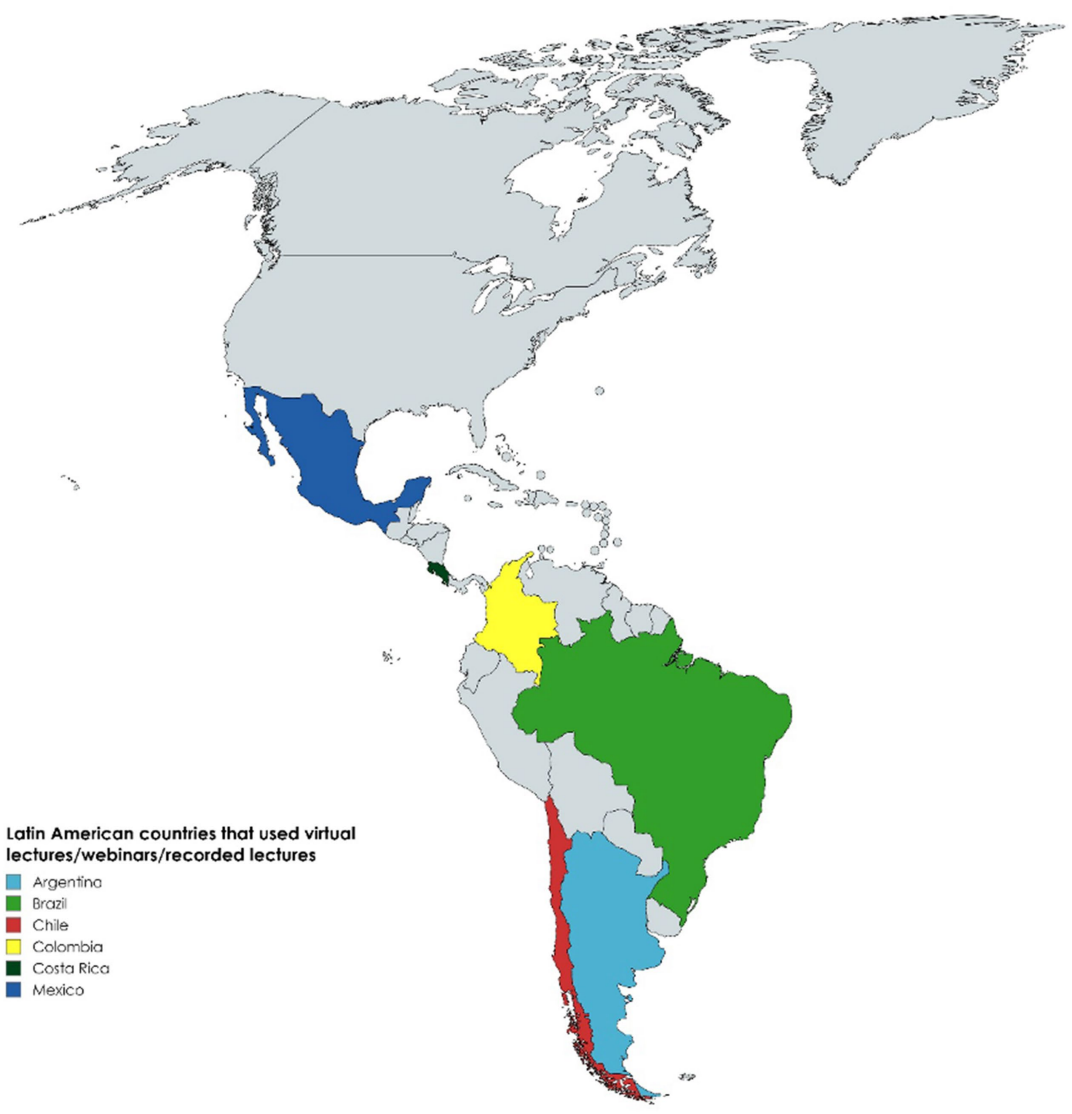
Latin American counties that used virtual lectures, webinars or recorded lectures.

The most common use of digital tools was for education, including online lectures, informative webinars, information via social media, pre-recorded video lectures, online evaluations, and clinical case webinars (Table 2) (2, 5–8, 11, 12, 18, 20, 22, 24, 26, 30, 33, 35). Among all the surveys, most residency programs replaced face-to-face education with online courses, including neurosurgery residents in Spain and Latin America (87.1%), orthopedic residents in Chile (86%), ophthalmologic residents in Brazil (95%), head and neck surgery residents in Brazil (78.3%), general surgery residents in Mexico (82.4%), otorhinolaryngology residents in Chile (87%), and gynecology residents in Brazil (85%) (5, 6, 8, 17, 18, 22, 26). Figure 3 shows a geographical representation of the countries that reported implementing changes in lectures during the COVID-19 pandemic.

**Table 2:** Consequences of the COVID-19 pandemic in surgery residency.

Program	Country	Decrease in surgeries per resident	Decrease of in person lectures	Decrease in hospital attendance	Service shift to COVID-19 areas	Residents sent to preventive quarantine	Lack of access to virtual tools
Alvarez M, 2020	Chile, Santiago de Chile	x	x	x	x	x	
Alvarez M, 2021	Chile, Santiago de Chile	x	x	x		x	
Arenas-Soto, 2021	Colombia	x	x	x			x
Brito L, 2020	Brazil	x	x		x		
Cabrera L, 2020	Colombia	x	x	x			
Chatziralli I, 2020	South America		x				
Cho M, 2020	South America						x
De la Cerda-Vargas M, 2021	Mexico, Brazil, Colombia, Argentina, Spain	x	x	x	x		
De la Cerda-Vargas M, 2022	Mexico, Argentina, Brazil, Colombia	x	x		x		
Carlos E. Díaz-Castrillón, 2021	Colombia					x	
Diaz Castrillón C, 2020	Colombia	x			x		
Falcioni A, 2022	Argentina, Buenos Aires	x					
Figueroa F, 2020	Chile, Santiago de Chile	x	x	x			
Gondim M, 2021	Brazil		x	x	x		
Gonzales-Urquijo M, 2021	México, Monterrey		x				
Gorgen A, 2021	Brazil, Porto Alegre	x		x	x		
Huamanchumo-Suyon M, 2020	Peru, Lima	x					
Leite Ana Kober, 2021	Brazil	x	x				
Lerendegui Luciana, 2021	Argentina, Buenos Aires	x			x	x	
Lima D, 2020	South America						
Munjal Tina, 2020	Worldwide (37.5% South Amercia)		x		x		x
Oropeza-Aguilar Mariano, 2022	Mexico, Mexico City	x		x	x		
Paesano Nahuel, 2020	Latin America	x			x		
Pagotto Vitor, 2020	Brazil, São Paulo	x			x		
Palacios Huatuco René, 2021	Argentina	x		x	x	x	
Pawlak KM	South America	x					
Prezotti, 2021	Brazil	x	x	x			
Rivera-Chavarría, 2021	Costa Rica		x	x			
Rodriguez Santos, 2021	Argetnina	x	x	x		x	
Santos, 2021	Argentina, Buenos Aires	x	x	x			
Trujillo, 2021	Peru, Lima	x					
Total		24	17	14	13	6	3

This frequent use of virtual courses brought an increase in academic activities; for example, in Argentina, there was an increase of 25 to 75% in academic activities, and in Brazil, an increase in the frequency of weekly seminars was reported (20, 35). Also, in Costa Rica, trainees who had once-a-week lectures before the pandemic, were later subjected to five lectures per week (a 400% increase) (31). Residents not only had more time to focus on academic activities provided by their university, but they were also able to attend webinars from other institutions and actively participate in medical social media platforms. A survey about social media use reported that the preferred platforms used to acquire information were YouTube, Whatsapp, and Zoom for webinars (24).

However, class hours were considerably reduced in some programs. De la Cerda-Vargas et al., reported that before the pandemic, neurosurgery residents in Latin America and Spain accumulated over 70 h per week of lectures, and during the pandemic these were reduced by 12% (12). Ophthalmologists that were surveyed mentioned similar numbers, with 26.2% of residents spending no more than 1-3 h in academic activities (10). The distribution of lecture hours differed between programs, with trainees reporting increased hours instead of reductions during the start of the pandemic. A survey based in Argentina mentioned that in the early pandemic 95% of trainees saw increased time in academic activities, with 82% of them spending more than 3 h per day. This study also showed that while they experienced increased hours, these later dropped 27% (33).

A Brazilian survey based on head and neck surgeons, showed that 78.3% (*n* = 36) considered that lectures were successfully adapted to online platforms (22). However, one resident experienced total interruption of all educational activities, 16 residents noted a 25% decrease, and 9 residents reported a moderate to severe reduction (22). Similar adaptations were seen with urology residents in Brazil, where a lot of teaching methods differing from lectures (such as grand rounds, conferences, and bedside teaching) were canceled and had to be replaced with an online equivalent (30). In this case, residents preferred virtual and online courses as their primary source of education. However, two studies reported major problems in virtual learning; 53.6% of Brazilian urology residents requested postponing the finishing date of their residency program, and 20.8% suggested additional training (30). On the other hand, obstetrics and gynecology residents saw that while 85% of their programs had online courses, 95% of them did not include surgical skill training for residents (8).

As expected, in-person conferences were severely reduced in most cases and were in turn replaced by virtual lectures. Surgical residents in Mexico City reported that 82.4% transitioned to distance learning (26). Urology trainees from Ibero-America also switched to virtual lectures (2). Both studies used similar platforms like Zoom, Skype, and Google-Meet. Ophthalmology residents also reported using Zoom (10). Other studies used prerecorded lectures that were published on their website and did not use real-time teaching with teleconferencing software (5, 8).

In some cases, the reduction of lecture-time resulted in more time dedicated to research. Argentinean residents saw an overall increase in research: on a first survey, 71% declared to be involved in 2 or more research studies, and 6 months later, 82% finished or published at least 1 of them (33). Similarly, 53% of residents surveyed in Mexico City stated that they used their time toward research activities (26).

### Complementary activities

Several programs installed and developed various activities to complement residents’ formation due to the lack of regular practice. The activities reported were simulation, virtual conferences, social media medical content, research opportunities, and surgical case discussions. The reported consequences of the COVID-19 pandemic in surgery residency programs and the compensatory measures implemented by each are summarized in Tables 2, 3, respectively.

**Table 3:** Compensatory activities implemented by the surgical programs.

Program	Country	Telemedicine	Virtual lectures	Outsourced Webinars	Social Media	Pre-recorded video lectures	Clinical Case webinars	Simulation training	Research Opportunities	Psychological support
Alvarez M, 2020	Chile, Santiago de Chile		x					x	x	x
Alvarez M, 2021	Chile, Santiago de Chile		x			x	x	x	x	
Arenas-Soto, 2021	Colombia	x	x							
Brito L, 2020	Brazil		x					x		
Cabrera L, 2020	Colombia		x		x		x	x	x	
Chatziralli I, 2020	South America		x	x		x	x			
Cho M, 2020	South America		x			x				
De la Cerda-Vargas M, 2021	Mexico, Brazil, Colombia, Argentina, Spain		x			x		x		
De la Cerda-Vargas M, 2022	Mexico, Argentina, Brazil, Colombia									
Carlos E. Díaz-Castrillón, 2021	Colombia	x	x							
Diaz Castrillón C, 2020	Colombia									
Falcioni A, 2022	Argentina, Buenos Aires							x		
Figueroa F, 2020	Chile, Santiago de Chile		x	x			x	x		
Gondim M, 2021	Brazil		x							
Gonzales-Urquijo M, 2021	México, Monterrey		x				x			
Gorgen A, 2021	Brazil, Porto Alegre							x		
Huamanchumo-Suyon M, 2020	Peru, Lima									
Leite Ana Kober, 2021	Brazil		x							
Lerendegui Luciana, 2021	Argentina, Buenos Aires							x		
Lima D, 2020	South America		x	x	x	x				
Munjal Tina, 2020	Worldwide (37.5% South Amercia)		x				x	x		
Oropeza-Aguilar Mariano, 2022	Mexico, Mexico City	x	x						x	
Paesano Nahuel, 2020	Latin America	x	x	x	x	x				
Pagotto Vitor, 2020	Brazil, São Paulo									
Palacios Huatuco René, 2021	Argentina		x							
Pawlak KM	South America									x
Prezotti, 2021	Brazil		x	x		x	x			
Rivera-Chavarría, 2021	Costa Rica		x			x				
Rodriguez Santos, 2021	Argetnina		x	x				x	x	x
Santos, 2021	Argentina, Buenos Aires		x						x	
Trujillo, 2021	Peru, Lima				x			x		
Total		4	22	6	4	8	7	12	7	3

### Simulation

We found that 16.13% (*n* = 5) of the programs in our review reported the use of different simulation systems as a way of maintaining the quality of surgical training (23, 26, 30, 33, 34). Due to the pandemic, the application of surgical simulation was also a challenge because, traditionally, simulation has been conducted in-person with tutors directly assessing residents in a skills lab; therefore, during the pandemic, surgical simulation had to shift to a remote format, which was facilitated through virtual platforms such as Zoom meetings and virtual real-time hands-on practice validated with Global Operative Assessment of Laparoscopic Skills (GOALS) and Fundamentals of Laparoscopic Surgery (FLS) criteria and assessed by a certified surgeon (16). This was the most common used methodology and was used by Peru and Argentina (16). Additionally, there was an important increase in time dedicated to surgical simulation. Before the pandemic, students had 1 h of training per month, compared to the pandemic where residents reached more than 20 h of simulation per month (23, 26). However, this kind of training did not provide confidence on surgical skills as 70% of the residents from a pediatric surgery training program from Argentina reported a decrease in their surgical confidence even when receiving simulation training (23). Since remote simulations had to be conducted at home or during the residents’ free time, such practices lacked the support of a trained evaluator to guide them, resulting in low adherence (23). For example, a study analyzing data from 18 surgical training programs in Mexico reported that only 41% of residents were using the virtual simulators consistently (26).

### Satisfaction of residents

Several examined programs assessed resident satisfaction with the modifications implemented during the pandemic. Six programs investigated residents’ perceptions of their surgical skills during the pandemic, and all surveys yielded unfavorable outcomes (21, 22, 29, 30, 35). Furthermore, a Brazilian study on urology residents specified that more advanced residents (PGY4 and 5) estimated a greater damage than PGY3; in some cases, several residents suggested prolonging their residency for one more year before graduating to supplement their perceived deficiencies (21, 30).

Contrarily, of the six studies that asked about the resident’s opinion over virtual lectures, most answers were positive (7, 10, 16, 17, 19, 26); 68.9% had better attendance to lectures than before the pandemic (19, 26) and 57% (*n* = 514) of respondents from three different programs, had positive opinions regarding virtual lectures, webinars, and conferences (7, 10, 26). A cross-sectional study from Chile that compiled the answers from 100 orthopedic residents on 7 different training programs across the country revealed that 82% of respondents would continue attending virtual conferences even after the pandemic restrictions stop (17). The interviewed residents judged different forms of education and rated webinars and virtual presentations very highly. In fact, when asked which online-based educational instances would they continue to use after the pandemic, none (*n* = 0) of the residents would continue watching surgical videos, while 75% (*n* = 75) considered webinars and presentations were worth watching after restrictions are lifted.

Additionally, only two programs asked about the resident’s satisfaction over the use of simulators; 68% (*n* = 42) of respondents from the study by Falcioni et al., that reports answers from residents, fellows, and staff from different institutions from Argentina, Brazil, Costa Rica, Guatemala, and Peru, said their laparoscopic simulation training experience was excellent, and 32% (*n* = 20) said it was good (16). On the contrary, in the Lerendegui et al. study, 70% (*n* = 7) of residents who went through simulation-based surgical training (mostly laparoscopic simulators) reported that their surgical skill confidence was reduced (23).

Overall, the findings suggest that in all our included Latin-American surgical programs, and across various surgical specialties, while efforts were made to compensate for the lack of real-patient interaction, most residents reported a negative impact of such efforts in their surgical skills but were fond of the implementation of virtual conferences.

## Discussion

The impact of the COVID-19 pandemic was felt worldwide, affecting several aspects of medicine, in particular, medical education (37). Education could no longer be delivered in person, causing several repercussions; residents developed deficiencies during their specialty training, and even during the selection process for residency, existing bias against minorities was exacerbated (37). These are just a few examples of the way the pandemic impacted medical education, especially in underdeveloped countries. Therefore, our review analyzed several aspects of surgical residency to determine how education was compromised in Latin American countries and the strategies that were used to overcome this. We identified that the pandemic affected surgical training (causing a negative impact on surgical skill), caused reassignments to treat COVID-19 patients, forced residents to implement telemedicine, forced programs to implement virtual training (including lectures, webinars, and simulation), and, in some cases, provided opportunities for other academic endeavors (such as research).

### Impact in surgical training and shift reassignments

In our study, 42% (*n* = 13) of programs reported sending their surgical residents to COVID-19 areas. Interestingly, during other pandemics such as cholera or the Spanish influenza, no data reporting a percentage of health workers reassigned to other areas, much less a specific number of surgical residents relocated, was found; however, the need to mobilize medical resources for the management of patients during those pandemics is described (38, 39). In the case of cholera, most patients required hospitalization for the administration of aggressive intravenous rehydration and antibiotics, which represented the use of hospital resources and space for prolonged periods of time, and the need for medical personnel to attend to this specific pathology, as seen during the COVID-19 pandemic (38). The Spanish influenza pandemic of 1918 is another example of the mobilization of medical resources, which resulted in nurses, medical officers, and other health care workers implementing public health measures to control the spread of the disease, leaving their usual activities to focus on community surveillance and health education (39).

In contrast to other pandemics, in our review we found a re-allocation of surgical residents to COVID-19 areas, resulting in a reported decrease in surgeries per resident in 77.4% (*n* = 23) of the programs; examples of similar changes in surgeries during other pandemics are difficult to find. An article describing neurosurgery during the Spanish influenza reported that the investigative work of important figures in the field of neurosurgery was affected; 30% of the hospital staff where Walter Dandy performed craniotomies was contaminated with influenza, which affected the number of patients he could treat during this period (40). Both Harvey Cushing and George Heuer had to treat cases of influenza because of the large number of patients, so they put their work as neurosurgeons on hold and performed fewer surgeries (40).

These examples illustrate the impact that pandemics have had on medical progress throughout human history, and how vast medical resources had to be used to treat the large number of patients affected. Therefore, it’s not unsurprising to see that a similar effect was seen during the COVID-19 pandemic in our study.

### Telemedicine

Our study showed that telemedicine was used to provide effective attention to patients in 12% (*n* = 4) of the programs included, which allowed residents to practice surgical follow-up despite the restrictions caused by the pandemic– a benefit that was possible thanks to the technological advancements of the 20th century. In the latest decade, the usage of technology in medicine has been increasing exponentially; online health consultation has largely reduced the need for person-to-person interviews and is now seen as an effective and cheap model for healthcare institutions (41–43). In China, “In Ping An Good Doctor,” one of its biggest online consultation services, was rated 5 out of 5 by most users, indicating satisfaction; therefore, Pan et al. suggested that telemedicine should be used as a complement to traditional medicine and only used to diagnose and treat diseases that prove to be uncomplicated and common (41). Telemedicine could also be useful not only for diagnosing diseases but also for controls in pregnant women as antenatal care, although it may be a dubious method for some of these patients; this method can provide relevant information to patients that they may otherwise find online but with a higher risk of bias (42). Finally, in the field of oncology, online consultation during the COVID-19 pandemic was found to be beneficial for patients that have limited economic resources and have major physical disabilities, but was also met with some resistance by patients that struggle to overcome unfamiliarity to these tools (43).

### Changes in lectures

In our study, lectures suffered significant changes, like the reduction of hours due to the restriction of person-to-person interaction, which resulted in the need to replace these methods with an online equivalent or other type of academic activities such as research. The current goal of online medical training is optimization so that it can be safely implemented into the curriculum of medical students and residency programs; a 1998 study from the United States found that surgery residents that received computer-based lectures had less skill proficiency than students that received normal lectures (44). However, online-based learning had a major advantage because it did not allow residents to break down a particular skill and help the student reinforce motions that were more troublesome than others (44). Another study that taught gross anatomy with video clips found that students that saw them had lower academic scores than those who attended face-to-face dissections (45). There was a lower level of satisfaction in students who learned with multimedia; this study suggests that hands-on training is better because it has more cognitive stimuli that can contribute positively to learning gross anatomy, which is fundamental in a surgical context (45).

Other authors argue that hands-off learning, with multimedia, should be aimed at improving specifical downfalls in surgical knowledge, but that these resources should follow strict standardization to provide assurance of quality and peer review; this often does not happen in YouTube or other commonly used sources by students, where the quality of the video is dependent on the creator, who may not always be trustworthy and up to medical standards (46). Residency programs that aim to improve audiovisual teaching methods should have a robust system of script writing, recording, and video development, which can be a problem according to an Australian study; they also discussed that producing a surgical video can represent various ethical problems and issues with intellectual property (47). Despite these problems, the American College of Surgeons developed e-learning clinical scenarios that have been implemented across residency programs. This tool is helpful in providing adequate and complete training to residents, and a similar approach has also been adopted by the European Association of Cardio-Thoracic Surgery (48). These resources can help provide adequate education toward surgery trainees across most residency programs.

Our study showcases that the pandemic resulted in major foundational remodeling of Latin American surgical programs, taking into account administrative, pedagogical, social, and technical issues. Despite the negative perceptions of residents detected in our study, technology and the capacity to implement fast solutions enabled the residents to continue their surgical education (with some downfalls), a benefit that was not available in previous pandemics. Newer studies should focus solely on applying pedagogic techniques that allow online teaching to the point where online medical education can be efficient and trustworthy in Latin American medical schools.

### Impact of online education

The results of our study show that surgical residents reported the highest attendance at virtual conferences/lectures, and the majority had positive opinions. This could be explained by the fact that the biggest complementary activity implemented during the pandemic was the attendance to virtual conferences and lectures, which take less resources both to hold and to attend (making attending easier than on-site lectures). In fact, 48.38% of the programs reviewed (*n* = 15) commented on the use of virtual lectures, but only 16.12% (*n* = 5) reported using or promoting webinars held by other institutions.

There is a great deal of literature from the last few years that compares the impact of in-person versus virtual conferences. The main limitation of virtuality is the lack of interaction between the expositor and the participants and between the participants themselves (49, 50). Mair et al. defined three key positive factors that make in person conferences better: (1) The participation produces the feeling of belonging to a community; (2) the discussion of a topic leads to new perspectives and new developments; and (3) meetings with peers can increase satisfaction and, consequently, increase performance (50–52).

However, virtual conferences improved overall accessibility by reducing limitations regarding geographic location, financial cost, and time, which are key points in low-middle income countries (LMIC) (49, 50). Additionally, virtual conferences allow to reduce academic and cultural discrimination, and, as Sardelis et al. proposed, it is a major factor to consider in evaluating the overall quality of a conference (53). Nevertheless, practice-based teaching (PBT) has been proven to be a useful tool to cope with the lack of interaction and overall learning in virtual activities (54). According to reports, PBT courses have proven beneficial in developing crucial job-related abilities such as problem-solving, leadership, teamwork, and practical skills that can be applied in the workplace (54).

The academic shift toward virtuality has been one of the biggest effects of the pandemic in modern medicine. The fact that some residents commented that they had acquired a habit of attending webinars and will continue to attend after the social distancing limitations are suspended is surprising. Further observation is going to be necessary to better characterize this phenomenon of virtuality in surgical education.

### Implementation of simulators

A simulator is a tool that reproduces one or more aspects of the working environment to prepare and train a student for a real-life situation (55). In our study, we found discordant results regarding the satisfaction of residents that had laparoscopic training through simulation (16, 23). As a matter of fact, from the two Argentinean reports that analyzed self-perception of surgical skills after using virtual simulators, one program reported almost exclusively positive opinions, in contrast with the other program that reported a negative impact in 70% of the residents (16, 23).

This could be explained by the lack of high-fidelity simulators. As Persoon et al. stated, low fidelity simulators are only useful in “novices” (for example, in pre-clinical students, a simple, low-cost suturing simulator is a well-fitted tool that stimulates and promotes learning) (56). However, in more advanced trainees, a higher fidelity simulator is required to ensure optimal learning (for example, a virtual reality simulator that recreates the clinical environment) (56). Studies have shown that creating a realistic setting in advanced simulators can enhance the psychological and emotional impact of the simulation; proportionally, the absence of realism is linked to a lower stimulus for the students, and therefore, less impact and quality of learning (57). Additionally, it is important to note that the biggest limitations of implementing useful simulators for training are the elevated cost of high-fidelity simulators, training infrastructure, and trained instructors, which significantly affects their proper implementation in LMIC (58).

Nevertheless, other resources have demonstrated to be useful, such as live interaction between the trainees and the trainer (59). A study performed by Diaz-Gio et al., including medical students and anesthesia residents from Mexico, Colombia, and Ecuador, demonstrated that online-synchronized simulation with immediate feedback and interaction is an effective and affordable tool to enhance virtual simulation (59). The major weaknesses reported in this model were technical problems such as unstable internet connections, the impossibility of simultaneous speaking, and the insufficient practice of motor skills.

### Limitations

As seen on Table 1, one of the potential limitations of our review is the inclusion of articles graded as high risk of bias according to our methodology and to the application of the National Heart, Lung, and Blood Institute (NHLBI) Study Quality Assessment Tools (4). This could be due to the context and design of the literature reviewed; most articles analyzed included qualitative cross-sectional surveys that lacked comparison between groups pre-and post-pandemic data, which limited the ability to conduct proper statistical analyses. This resulted in a high risk of bias according to the tool we applied. Furthermore, results from surveys measuring self-perception about any topic will have several limitations including the generalizability of results as well as their reproducibility. Nonetheless, as our objective was to characterize these self-perceptions as an outcome of the deleterious effect that the pandemic had on surgical education, we believe that the measured bias does not significantly affect the main conclusions and findings of our review.

## Conclusions and future directions

The COVID-19 pandemic significantly disrupted surgical education in Latin American countries, leading to the reallocation of resources, including surgical residents, to non-surgical duties such as COVID-19 patient care. This reassignment affected surgical training, reducing the number of surgeries performed by residents and prompting a shift to virtual learning methods like telemedicine, online lectures, and webinars. While virtual education enabled continuity in training, it posed challenges in terms of interaction and skill acquisition, with some residents expressing dissatisfaction, particularly with low-fidelity simulators. The pandemic also highlighted resource limitations in Latin American countries, making it difficult for hospitals and universities to quickly adapt to online teaching models and advanced simulators due to financial and technical constraints. Despite these challenges, many programs were able to innovate and continue providing education through virtual platforms.

Having this in consideration, we recommend that universities and teaching hospitals develop hybrid curricula that include a database of online lectures that follow the same quality of conventional lectures to be used in a situation similar to the COVID-19 pandemic and ensure continuing education of their residents; it’s important to note that this strategy can be applied even in low resource settings. Furthermore, we advise against suspending surgical practices, as simulation in resource limited setting has not been developed enough to replace conventional surgery; furthermore, a hands-on experience, taking every precaution, can be more cost effective than implementing simulation protocols across all curricula. As medical education continues to evolve, there is a need for further research and investment in pedagogical techniques that enhance online learning and simulation in surgery, particularly in low-resource settings, to ensure that the next generation of surgical residents is adequately trained. Therefore, we advocate for the creation of new policies that take these points into consideration, particularly looking at easily implemented cost effective solutions to ensure the continuity of medical education in low-income settings in future pandemics or major world-wide disruptions.

## Data Availability

The original contributions presented in the study are included in the article/Supplementary material, further inquiries can be directed to the corresponding author.
