# Supplementary material for: Impact of the COVID-19 pandemic on surgical education and training programs in Latin America: a systematic review
Source: Front Med (Lausanne). 2024 Nov 20;11:1499436. doi: 10.3389/fmed.2024.1499436 (PMC11614603; doi:10.3389/fmed.2024.1499436)
Supplement: Supplementary file 1 [file Table_1.DOCX]

**SUPPLEMENTARY MATERIAL 1**

Search Strategies:

**PubMed:** ((surgical training) OR (medical training) OR (tele-education) OR (telemedicine) OR (virtual learning) OR ("Internship and Residency"[Mesh])) AND ((covid) OR (Covid 19 pandemic) OR (pandemic) OR (COVID) OR (Sars Cov 2) OR ("COVID-19"[Mesh])) AND ((Latin America) OR (South America) OR ("Latin America"[Mesh]) OR (Belize) OR (Costa Rica) OR (El Salvador) OR (Guatemala) OR (Honduras) OR (Mexico) OR (Nicaragua) OR (Panama) OR (Argentina) OR (Bolivia) OR (Brazil) OR (Chile) OR (Colombia) OR (Ecuador) OR (Guyana) OR (Paraguay) OR (Peru) OR (Suriname) OR (Uruguay) OR (Venezuela) OR (Cuba) OR (Dominican Republic) OR (Haiti) OR (Trinidad y Tobago) OR (LMICS))

**VHL:** (residencia OR residency OR (surgical training) OR (entrenamiento quirúrgico) OR (medical training) OR (entrenamiento medico) OR tele-education OR telemedicine OR telemedicina OR (virtual learning) OR (entrenamiento virtual)) AND (( mh:("COVID-19")) OR covid OR (Covid 19 pandemic) OR SARS-CoV-2) AND ((latin america) OR (américa Latina) OR latinoamerica OR (South America) OR sudamérica OR Belize OR Belice OR (Costa Rica) OR (El Salvador) OR Guatemala OR Honduras OR Mexico OR Nicaragua OR Panama OR Argentina OR Bolivia OR Brazil OR Chile OR Colombia OR Ecuador OR Guyana OR Paraguay OR Peru OR Suriname OR Uruguay OR Venezuela OR Cuba OR (Dominican Republic) OR Haiti OR (Trinidad y Tobago) OR (Trinidad and Tobago) OR LMICS)

**Scopus:** TITLE-ABS-KEY ( ( residency OR ( surgical AND training ) OR ( medical AND training ) OR tele-education OR telemedicine OR ( virtual AND learning ) ) AND ( covid OR ( covid 19 pandemic ) OR sars-cov-2 ) AND ( ( latin AND america ) OR ( south AND america ) OR belize OR ( costa AND rica ) OR ( el AND salvador ) OR guatemala OR honduras OR mexico OR nicaragua OR panama OR argentina OR bolivia OR brazil OR chile OR colombia OR ecuador OR guyana OR paraguay OR peru OR suriname OR uruguay OR venezuela OR cuba OR ( dominican AND republic ) OR haiti OR ( trinidad AND y AND tobago ) OR ( trinidad AND tobado ) OR lmics ) )
